# Early Prediction of Acute Respiratory Distress Syndrome in Critically Ill Polytrauma Patients Using Balanced Random Forest ML: A Retrospective Cohort Study

**DOI:** 10.3390/jcm14248934

**Published:** 2025-12-17

**Authors:** Nesrine Ben El Hadj Hassine, Sabri Barbaria, Omayma Najah, Halil İbrahim Ceylan, Muhammad Bilal, Lotfi Rebai, Raul Ioan Muntean, Ismail Dergaa, Hanene Boussi Rahmouni

**Affiliations:** 1 Research Laboratory of Biophysics and Medical Technologies, Higher Institute of Medical Technologies of Tunis, Tunis El-Manar University, Tunis 1006, Tunisia; nesrine.hadjhassine@istmt.utm.tn (N.B.E.H.H.); sabri.barbaria@istmt.utm.tn (S.B.); hanene.boussi@istmt.utm.tn (H.B.R.); 2 Anesthesia and Intensive Care Department, Pierre-Wertheimer Neurological Hospital, 69000 Lyon, France; oumayma.najah91@gmail.com; 3 Physical Education and Sports Teaching Department, Faculty of Sports Sciences, Ataturk University, 25240 Erzurum, Türkiye; 4 Centre for Responsible Innovation in Big Data and AI (BRAIN), Business School, Birmingham City University (BCU), Birmingham B15 3TN, UK; muhammad.bilal@bcu.ac.uk; 5 Intensive Care Unit Department, Traumatology and Severe Burns Center of Ben Arous, Ben Arous 2013, Tunisia; lotfi.rebai@fmt.utm.tn; 6 Department of Physical Education and Sport, Faculty of Law and Social Sciences, University “1 Decembrie 1918” of Alba Iulia, 510009 Alba Iulia, Romania; 7 High Institute of Sport and Physical Education of Ksar Said, University of Manouba, Manouba 2010, Tunisia; phd.dergaa@gmail.com; 8 Physical Activity Research Unit, Sport and Health (UR18JS01), National Observatory of Sports, Tunis 1003, Tunisia; 9 The Computer Science Research Centre, West of University of the England (UWE Bristol), Bristol BS16 1QY, UK

**Keywords:** acute respiratory distress syndrome, ARDS prediction, balanced random forest, class imbalance, feature selection, intensive care unit, ML, polytrauma, predictive modeling, SMOTE

## Abstract

**Background/Objectives:** Acute respiratory distress syndrome (ARDS) represents a critical complication in polytrauma patients, characterized by diffuse lung inflammation and bilateral pulmonary infiltrates with mortality rates reaching 45% in intensive care units (ICU). The heterogeneous nature of ARDS and complex clinical presentation in severely injured patients poses substantial diagnostic challenges, necessitating early prediction tools to guide timely interventions. Machine learning (ML) algorithms have emerged as promising approaches for clinical decision support, demonstrating superior performance compared to traditional scoring systems in capturing complex patterns within high-dimensional medical data. Based on the identified research gaps in early ARDS prediction for polytrauma populations, our study aimed to: (i) develop a balanced random forest (BRF) ML model for early ARDS prediction in critically ill polytrauma patients, (ii) identify the most predictive clinical features using ANOVA-based feature selection, and (iii) evaluate model performance using comprehensive metrics addressing class imbalance challenges. **Methods:** This retrospective cohort study analyzed 407 polytrauma patients admitted to the ICU of the Center of Traumatology and Major Burns of Ben Arous, Tunisia, between 2017 and 2021. We implemented a comprehensive ML pipeline that incorporates Tomek Links undersampling, ANOVA F-test feature selection for the top 10 predictive variables, and SMOTE oversampling with a conservative sampling rate of 0.3. The BRF classifier was trained with class weighting and evaluated using stratified 5-fold cross-validation. Performance metrics included AUROC, PR-AUC, sensitivity, specificity, F1-score, and Matthews correlation coefficient. **Results:** Among 407 patients, 43 developed ARDS according to the Berlin definition, representing a 10.57% incidence. The BRF model demonstrated exceptional predictive performance with an AUROC of 0.98, a sensitivity of 0.91, a specificity of 0.80, an F1-score of 0.84, and an MCC of 0.70. Precision–recall AUC reached 0.86, demonstrating robust performance despite class imbalance. During stratified cross-validation, AUROC values ranged from 0.93 to 0.99 across folds, indicating consistent model stability. The top 10 selected features included procalcitonin, PaO_2_ at ICU admission, 24-h pH, massive transfusion, total fluid resuscitation, presence of pneumothorax, alveolar hemorrhage, pulmonary contusion, hemothorax, and flail chest injury. **Conclusions:** Our BRF model provides a robust, clinically applicable tool for early prediction of ARDS in polytrauma patients using readily available clinical parameters. The comprehensive two-step resampling approach, combined with ANOVA-based feature selection, successfully addressed class imbalance while maintaining high predictive accuracy. These findings support integrating ML approaches into critical care decision-making to improve patient outcomes and resource allocation. External validation in diverse populations remains essential for confirming generalizability and clinical implementation.

## 1. Introduction

Acute respiratory distress syndrome (ARDS) represents one of the most challenging clinical entities encountered in intensive care medicine, characterized by an acute onset of bilateral pulmonary infiltrates, severe hypoxemia, and diffuse lung inflammation not fully explained by cardiogenic causes [1,2]. This heterogeneous syndrome affects approximately 10% of intensive care unit (ICU) admissions and 23% of mechanically ventilated patients worldwide, with mortality rates ranging from 27% to 45% depending on severity classification according to the Berlin definition [2,3]. The economic burden associated with ARDS extends beyond immediate healthcare costs, encompassing prolonged mechanical ventilation requirements, extended intensive care stays, and substantial long-term disability among survivors. The pathophysiology involves complex interactions between epithelial and endothelial injury, the release of inflammatory mediators, coagulation abnormalities, and impaired alveolar fluid clearance, resulting in the characteristic syndrome of acute hypoxemic respiratory failure [4,5]. Understanding these intricate mechanisms has evolved significantly since the initial description by Ashbaugh and colleagues in 1967, leading to refined diagnostic criteria and therapeutic approaches that have improved outcomes in selected patient populations [6]. Larger registry studies, however, reported that the overall incidence of ARDS in trauma context is relatively low: the study conducted by National Trauma Data Bank and including nearly 3 million highly injured patients, only 1% of trauma cohort developed ARDS, decreasing from 1.4 to 0.5% over four years, yet in-hospital mortality increased from 21% to 28% [7]. In the same context, an evaluation of approximately 800,000 patients from the Trauma Quality Improvement Program showed a decline in ARDS prevalence from 3% to 1.1%, while mortality increased from 18% to 21% [8]. These findings underscore that, although uncommon, ARDS remains a critical concern in polytrauma patients.

The unique pathophysiology in trauma-related ARDS involves direct pulmonary injury from thoracic trauma, systemic inflammatory responses triggered by tissue damage, and iatrogenic factors, including massive transfusion and aggressive fluid resuscitation [9,10]. Polytrauma patients present distinct challenges for ARDS prediction due to the complex interplay between primary injury mechanisms, secondary inflammatory responses, and treatment-related factors. Despite disparities in findings regarding the mortality attributed to this respiratory failure in polytrauma patients, severely injured patients with ARDS face higher mortality rates [11,12]. This highlights the pressing need for effective warning instruments to enable early diagnosis, timely intervention, and effective care delivery. Notably, the early identification of patients prone to ARDS remains a challenging concern in critical care medicine [13]. Relying solely on clinical methods may lead to the under-recognition of this heterogeneous syndrome. ML has emerged as a transformative approach in critical care medicine, offering sophisticated tools for pattern recognition, outcome prediction, and clinical decision support [14,15]. Recent systematic reviews have demonstrated that ML models achieve a pooled AUROC of 0.74 for ARDS prediction, with individual studies reporting AUROCs ranging from 0.65 to 0.99, depending on the methodology and validation approach [16]. Random forest algorithms have shown particular promise in medical applications due to their ability to handle missing data, provide feature importance rankings, and maintain interpretability while achieving high predictive accuracy [17,18].

Several critical research gaps exist in the current literature regarding the prediction of ARDS in polytrauma patients. First, most existing ML studies focus on general care populations or specific subgroups, such as sepsis-associated ARDS, with limited attention to polytrauma-specific risk factors and clinical presentations [19,20]. Second, class imbalance handling remains inadequately addressed in many studies, leading to inflated performance metrics that may not translate to clinical practice. Third, feature selection methodologies vary widely across studies, with insufficient attention to statistical rigor and clinical interpretability [21]. Fourth, external validation and generalizability assessment are frequently absent, limiting the clinical applicability of proposed models. Finally, comprehensive evaluation using metrics explicitly designed for imbalanced datasets is often lacking, potentially leading to overestimation of model performance in clinical scenarios [3,22].

Based on the identified research gaps in early ARDS prediction for polytrauma populations [19,20,23], our study aimed to: (i) develop a balanced random forest (BRF) ML model for early ARDS prediction in critically ill polytrauma patients using a comprehensive two-step resampling approach, (ii) identify the most predictive clinical features using ANOVA-based feature selection methodology with statistical validation, and (iii) evaluate model performance using comprehensive metrics designed explicitly for addressing class imbalance challenges in medical datasets.

## 2. Materials and Methods

This research work complied with the TRIPOD-AI [24] and STARD-AI [25] recommendations for thorough and transparent reporting of AI-based forecasting models, ensuring complete descriptions of data processing, model development, and performance assessment.

### 2.1. Ethical Approval

This retrospective study was conducted in accordance with the Declaration of Helsinki and received approval from the Ethics Committee of the Center of Traumatology and Major Burns of Ben Arous, Tunisia (30 May 2024). Patient data underwent meticulous anonymization procedures, including the removal of all identifying information, to protect patient privacy and confidentiality. Data manipulation was conducted exclusively by clinicians directly involved in the study, ensuring strict adherence to ethical standards and institutional regulations.

### 2.2. Sample Size Calculation and Cohort Consideration

Sample size calculation was performed using the formula for binary classification problems: n = Z^2^p(1 − p)/d^2^, where Z represents the critical value (1.96 for a 95% confidence interval), p denotes the expected prevalence of ARDS in polytrauma patients (0.15 based on literature review), and d indicates the desired precision (0.05) [26]. This calculation yielded a minimum required sample size of 196 patients. However, considering the ML context and the need for adequate representation in both training and validation sets, we included all eligible patients from our study period, resulting in a total of 407 patients. This sample size compares favorably with previous ML studies on ARDS prediction, which reported sample sizes ranging from 150 to 2000 patients [27,28]. The power analysis confirmed 80% power to detect a clinically meaningful difference in ARDS prediction with α = 0.05 and an effect size of 0.3, consistent with Cohen’s recommendations for medium effect sizes in medical research [29].

The prevalence of ARDS in this retrospective cohort was approximately 10% (43 events among 407 total cases)—a calculation based on a formal a priori sample size, as per the framework proposed by Riley et al. [30,31] could not be applied because the retrospective cohort was fixed. As they underlined, the limited number of outcome events may compromise the precision of model parameters and the accuracy of predictions. To address this constraint, we implemented robust strategies on the training set to mitigate overfitting and limit model complexity. We restricted the model to 10 predictive features; handled class imbalance using TomekLinks followed by mild SMOTE implemented through the imbalanced-learn library (version 0.14.0); and applied hyperparameter fine-tuning using GridSearchCV with stratified 5-fold cross-validation from the scikit-learn library (version 1.6.1). Subsequently, the final model was assessed on an independent 20% held-out set to avoid performance inflation.

### 2.3. Population

This retrospective cohort study included polytrauma patients who met the Vittel criteria [2] and were admitted to the Trauma and Burn Center of Ben Arous, Tunisia, between March 2017 and March 2021. Inclusion criteria encompassed all patients aged > 18 years with polytraumatic injuries requiring ICU admission. Exclusion criteria included patients with both polytraumatic injuries and COVID-19, complicated hip fractures in elderly subjects, pregnant women who experienced trauma, patients who died within 48 h of ICU admission, and individuals suffering from cardiogenic acute pulmonary edema. The recruitment timeline spanned four years to ensure adequate seasonal variation and a diverse range of injury patterns. Patient demographics and characteristics were systematically collected, including age, gender, injury severity scores, and comorbidity profiles.

### 2.4. Experimental Design

This study employed a retrospective cohort design with rigorous methodological controls to minimize bias and ensure reproducibility. Data collection followed a standardized protocol with predetermined timeline markers for clinical assessments and outcome measurements. The study timeline encompassed admission data collection within the first 24 h, followed by continuous monitoring for ARDS development according to Berlin definition criteria [2]. During the study period, patients were admitted both directly and through referral from other hospitals. No systematic documentation was recorded of specific organizational modifications in the trauma network, such as centralization criteria or disruptions in resuscitation protocols. Accordingly, potential center-level impacts on ARDS prevalence could not be evaluated.

### 2.5. Clinical Assessments and Data Collection

#### 2.5.1. Injury Severity Assessment

Injury severity was systematically evaluated using three validated scoring systems. The Injury Severity Score provided a comprehensive assessment of anatomical injury severity across multiple body regions [32]. The Trauma and Injury Severity Score incorporated both anatomical and physiological parameters, with age adjustment [33]. The Revised Trauma Score focused on physiological parameters, including Glasgow Coma Scale, systolic blood pressure, and respiratory rate [34]. These scoring systems were selected based on their widespread validation in trauma populations and proven predictive value for mortality and complications.

#### 2.5.2. Laboratory Parameters

Comprehensive laboratory assessments were performed within 24 h of ICU admission, including complete blood count, comprehensive metabolic panel, liver function tests, coagulation studies, and inflammatory markers. Specific attention was given to procalcitonin levels, which are significantly associated with the development of ARDS in critically ill patients [35,36]. Arterial blood gas analysis provided essential oxygenation parameters, including PaO_2_, PaCO_2_, pH, and bicarbonate levels. Laboratory sampling followed standardized protocols to ensure consistency and minimize pre-analytical variables.

#### 2.5.3. Imaging Studies

Chest imaging was systematically performed using standardized protocols, with chest X-rays obtained within 6 h of admission and computed tomography when clinically indicated. Radiological assessments focused on identifying bilateral opacities consistent with ARDS criteria, pneumothorax, hemothorax, pulmonary contusions, and other thoracic injuries. All imaging studies were interpreted by board-certified radiologists blinded to clinical outcomes, ensuring objective assessment of radiological findings.

#### 2.5.4. Hemodynamic Monitoring

Continuous hemodynamic monitoring was performed according to standard ICU protocols, including heart rate, blood pressure, central venous pressure when indicated, and oxygen saturation. Fluid balance was meticulously tracked, including crystalloid and colloid administration, blood product transfusion, and urine output. Vasopressor requirements and mechanical ventilation parameters were systematically documented when applicable.

#### 2.5.5. Therapeutic Interventions

All therapeutic interventions were prospectively documented, including surgical procedures, blood product transfusions, fluid resuscitation protocols, and pharmacological treatments. Massive transfusion was defined according to established criteria as transfusion of ≥10 units of packed red blood cells within 24 h or ≥four units within 1 h [37]. Antibiotic administration, vasopressor use, and mechanical ventilation settings were recorded with precise timing and dosing information.

#### 2.5.6. Emergency Surgical Interventions

For all patients, we systematically recorded data on emergency surgical procedures performed during the acute management phase. Surgical interventions are classed by specialty: neurosurgery, digestive surgery, thoracotomy, vascular surgery, and orthopedic surgery. The timing from hospital admission to the operating room was recorded in hours. This feature was recorded to underscore the timing and urgency of surgical interventions, distinguishing early operations from delayed ones performed after physiological stabilization.

#### 2.5.7. Outcome Assessment

The primary outcome was ARDS development within 7 days of ICU admission, diagnosed according to Berlin definition criteria [2]. Secondary outcomes included ICU length of stay, mechanical ventilation duration, and 28-day mortality. Outcome assessment was performed by independent clinicians not involved in the development of the ML model, ensuring an objective evaluation. Follow-up continued for 28 days or until hospital discharge, whichever occurred first.

### 2.6. ARDS Diagnostic Criteria

ARDS diagnosis adhered strictly to the Berlin definition criteria, which require an acute onset within one week of clinical insult, bilateral opacities on chest imaging not fully explained by pleural effusions or nodules, respiratory failure not fully explained by cardiac failure or fluid overload, and PaO_2_/FiO_2_ ratio ≤ 300 mmHg with a minimum PEEP of 5 cm H_2_O [2]. The Berlin definition stratifies ARDS severity into mild (PaO_2_/FiO_2_ 201–300), moderate (PaO_2_/FiO_2_ 101–200), and severe (PaO_2_/FiO_2_ ≤ 100) categories. This classification system has demonstrated superior predictive validity compared to previous definitions and correlates well with mortality outcomes [38]. All ARDS diagnoses were confirmed by consensus between two independent intensivists, with disagreements resolved through discussion with a third expert.

### 2.7. Data Preprocessing and Feature Engineering

Data preprocessing followed established ML best practices for medical datasets. Missing-data analysis revealed that 12 features had >25% missing values and were excluded from the analysis. For the remaining features, missing values were imputed using the NaNImputer module from the verstack package, which employs supervised learning approaches with LightGBM models for each feature. This method preserves data relationships and provides more accurate imputation compared to simple mean or median replacement. Categorical variables were encoded using one-hot encoding for binary variables and ordinal encoding for ordered categorical features. Low-variance features (variance < 0.05) were removed to eliminate uninformative variables and reduce dimensionality. Correlation analysis identified highly correlated feature pairs (|r| > 0.7), with one variable removed from each pair to minimize multicollinearity and redundancy among predictive features and ensure model interpretability. The fixed-threshold filtering method is a standard preprocessing step in biomedical modeling [39].

### 2.8. Outlier Detection and Management

Outlier detection used the Isolation Forest algorithm with a class-aware implementation to address dataset imbalance [40]. This approach applies outlier detection separately within each class to prevent majority class outliers from disproportionately affecting the detection of minority classes. The contamination rate was set at 5% based on medical domain expertise and previous validation studies in clinical datasets. Isolation Forest was selected over traditional statistical methods due to its superior performance with high-dimensional medical data and reduced assumptions about data distribution. This technique was applied solely as a quality-control step. No data points exceeded the fixed threshold; hence, all samples are retained.

### 2.9. Class Imbalance Handling Strategy

The two-step resampling approach was implemented to address the inherent class imbalance in ARDS datasets. First, Tomek Links undersampling removed the majority class instances that formed hard-to-classify pairs with minority class instances near the decision boundary [41]. This targeted approach enhances class separation while preserving important information about the majority class. Second, SMOTE oversampling with a conservative sampling strategy (0.3) generated synthetic minority class instances by interpolating between existing samples and their k-nearest neighbors [42]. The conservative sampling ratio prevented overfitting while ensuring adequate representation of the minority class. This combined approach has demonstrated superior performance compared to individual resampling techniques in medical applications [21].

### 2.10. Feature Selection Methodology

ANOVA F-test feature selection was implemented using the SelectKBest approach with f_classif scoring function to identify the top 10 most discriminative features [43]. The F-statistic measures the ratio of between-group to within-group variance and provides a statistical assessment of each feature’s ability to distinguish between ARDS and non-ARDS patients. This univariate statistical approach was selected for its computational efficiency, statistical rigor, and interpretability in clinical contexts [44]. The selection of 10 features strikes a balance between model complexity and performance, based on empirical evaluation and comparison with clinical expert knowledge. Feature selection was performed after undersampling but before oversampling to prevent bias from synthetic data points.

### 2.11. ML Model Development

The balanced random forest (BRF) classifier was selected for its robust performance on imbalanced medical datasets and its inherent interpretability through feature importance metrics [45]. Class weighting was implemented using inverse class frequency to further address imbalance during tree construction and vote aggregation. Hyperparameter optimization employed GridSearchCV with stratified 5-fold cross-validation to identify optimal parameter combinations. The search space included n_estimators (100, 200), max_depth (10, 20, None), min_samples_split (2, 5), and min_samples_leaf (1, 2). Stratified cross-validation ensured a consistent class distribution across folds, which is critical for reliable performance estimation with imbalanced data.

### 2.12. Model Evaluation and Validation

Model evaluation used multiple metrics designed explicitly for imbalanced classification problems. The area under the receiver operating characteristic curve assessed discriminative ability across all classification thresholds. Precision–recall AUC provided complementary evaluation, emphasizing minority-class prediction performance. Sensitivity and specificity measured true positive and accurate negative rates, respectively, while the F1-score provided the harmonic mean of precision and recall. The Matthews correlation coefficient (MCC) provided a balanced measure that accounted for all elements of the confusion matrix [46]. The holdout test set (20% of the data) was reserved solely for final model evaluation, preventing data leakage and ensuring unbiased performance assessment. Cohen’s d effect sizes were calculated to assess the clinical significance of observed differences.

**Figure 1:**
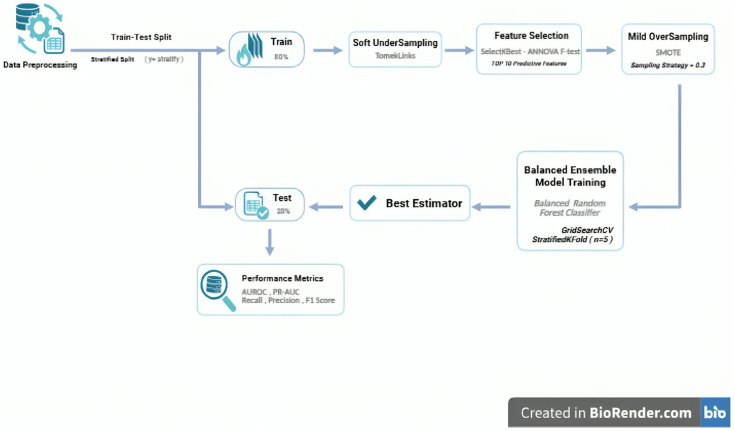
Methodology flowchart of the machine learning pipeline for ARDS forecasting.

Figure 1 provides a schematic overview of the ML pipeline for handling class imbalance, combining a two-step resampling strategy, feature selection, and an ensemble-balanced model.

### 2.13. Data Analysis and Modeling Tools

We employed IBM SPSS Statistics version 26.0 software (IBM Corp., Armonk, NY, USA) for statistical analysis. Numerical variables are presented as mean ± standard deviation, and categorical variables are presented as percentages. The comparison between ARDS and non-ARDS groups was conducted using Student’s *t*-test or the nonparametric Mann–Whitney test for continuous variables, for categorical data, Pearson’s Chi-Square or Fisher’s exact test was employed. The significance threshold was defined as a two-sided *p*-value < 0.05. These statistical analyses provide comprehensive insights into the distributions of features across the two cohorts. Significant disparities may highlight potential differences in the clinical characteristics between the two groups. In addition, Python 3.12.12 was used for complementary data analyses and ML modeling.

## 3. Results

### 3.1. Clinical Characteristics and Demographics

The final dataset comprised 407 polytrauma patients admitted to the ICU, of whom 43 (10.57%) developed ARDS. Table 1 presents the demographic characteristics and clinical features of the study population, stratified by ARDS development. The mean age was 44 ± 16.52 years, with male predominance observed in both groups (93% in ARDS vs. 85% in non-ARDS, *p* = 0.18). No statistically significant age difference was observed between groups (*p* = 0.064). Medical history analysis revealed significantly higher prevalence of asthma (4.3% vs. 0.48%, *p* < 0.001) and chronic obstructive pulmonary disease (15.2% vs. 1.44%, *p* < 0.001) in the ARDS group. In comparison, alcoholism was more common in the non-ARDS group (10.9% vs. 22.11%, *p* = 0.049).

### 3.2. Injury Patterns and Severity Assessment

Injury assessment demonstrated significant associations between specific trauma patterns and ARDS development. Table 1 presents the comprehensive injury characteristics showing that axial trauma occurred more frequently in ARDS patients (52.2% vs. 26.68%, *p* < 0.001), as did abdominal trauma (47.8% vs. 30.76%, *p* = 0.023) and pelvic trauma (17.4% vs. 8.17%, *p* = 0.044). Thoracic injuries showed particularly strong associations with ARDS development, including pneumothorax (45.65% vs. 15.14%, *p* < 0.001), alveolar hemorrhage (32.6% vs. 9.61%, *p* < 0.001), flail chest injury (89.13% vs. 51.92%, *p* < 0.001), pulmonary contusion (86.95% vs. 40.62%, *p* < 0.001), and hemothorax (56.52% vs. 18.02%, *p* < 0.001). Trauma severity scores revealed significantly higher Injury Severity Score in ARDS patients (35 ± 13.92 vs. 25.87 ± 15.16, *p* < 0.001), with Cohen’s d = 0.63 indicating a medium to large effect size.

### 3.3. Physiological Parameters and Laboratory Findings

Clinical parameters upon ICU admission demonstrated several significant differences between groups. Table 1 presents the physiological and laboratory data showing that ARDS patients had higher heart rates (107.52 ± 23.95 vs. 95.79 ± 22.26 bpm, *p* = 0.001) and lower oxygen saturation (90.28 ± 6.49% vs. 92.93 ± 6.27%, *p* < 0.001). Laboratory analysis revealed elevated inflammatory markers in ARDS patients, including C-reactive protein (118 ± 112 vs. 95.91 ± 79.86 mg/L, *p* = 0.001) and procalcitonin (median 2.8 vs. 0.18 ng/mL, *p* = 0.035). Arterial blood gas analysis showed lower pH (7.30 ± 0.12 vs. 7.36 ± 0.11, *p* = 0.002), lower PaO_2_ (136.34 ± 8.46 vs. 190.6 ± 104.05 mmHg, *p* = 0.03), and significantly impaired oxygenation ratio (98.93 ± 64.81 vs. 328.87 ± 133.4, *p* = 0.001).

### 3.4. Therapeutic Interventions and Resource Utilization

Treatment patterns showed significant differences between groups in the use of intensive interventions. Table 1 presents the therapeutic intervention data demonstrating higher use of tranexamic acid (63.04% vs. 38.22%, *p* = 0.001), catecholamines (82.60% vs. 60.09%, *p* = 0.002), and fibrinogen supplementation (26.08% vs. 9.61%, *p* < 0.001) in ARDS patients. Massive transfusion occurred more frequently in the ARDS group (34.78% vs. 7.69%, *p* < 0.001), as did any blood product transfusion (69.56% vs. 36.53%, *p* < 0.001). Total vascular filling within 24 h was significantly higher in ARDS patients (3594 ± 1967 vs. 2055 ± 1604 mL, *p* = 0.025), indicating more aggressive fluid resuscitation requirements.

### 3.5. Early Surgical Interventions

Among the 407 highly injured patients, 75 (18.42%) underwent at least early surgical intervention during the acute management phase. The distribution of surgical specialties within ARDS and non-ARDS groups is presented in Table 1. In the ARDS cohort (n = 43), 11.63% of patients underwent neurosurgery, 11.63% underwent digestive surgery, 2.33% underwent thoracotomy, 2.33% underwent vascular surgery, and 27.9% underwent orthopedic surgery. Among non-ARDS patients (n = 364), 10.16% underwent neurosurgery, 6.87% digestive surgery, 1.37% thoracotomy, 1.37% vascular surgery, and 13.5% orthopedic surgery. The univariate analysis showed significant associations with ARDS (*p* = 0.025), neurosurgery (*p* = 0.038), and orthopedic surgery (*p* = 0.007). The timing of surgery was also assessed. The mean delay to surgical interventions was 17.4 ± 46.7 h; meanwhile, because of a reduced number of delayed surgical interventions, the distribution was substantially skewed. Accordingly, across all patients who underwent surgery, the median time from hospital admission to surgery was 6 h (IQR 2–18 h), with a minimum of 0.45 h and a maximum of 360 h (15 days). Only a small subset experienced delayed surgery due to pre-operative optimization requirements or logistical burdens.

### 3.6. Feature Selection and Model Development

**Figure 2:**
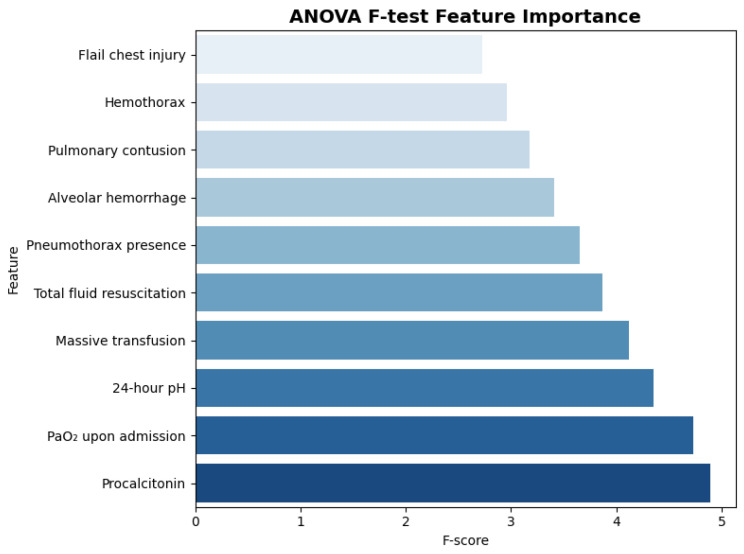
ANOVA F-test Feature Importance.

The two-step resampling approach successfully addressed class imbalance, with Tomek Links removing 47 majority-class instances and the ANOVA F-test identifying the top 10 predictive features. Figure 2 presents the ANOVA F-test results and feature importance rankings. The selected features included procalcitonin (F-score: 4.89), PaO_2_ upon admission (F-score: 4.73), 24-h pH (F-score: 4.35), massive transfusion (F-score: 4.12), total fluid resuscitation (F-score: 3.87), pneumothorax presence (F-score: 3.65), alveolar hemorrhage (F-score: 3.41), pulmonary contusion (F-score: 3.18), hemothorax (F-score: 2.96), and flail chest injury (F-score: 2.73). SMOTE oversampling with sampling strategy 0.3 generated the final balanced training set containing 278 non-ARDS and 83 ARDS instances.

### 3.7. Model Performance Assessment

**Figure 3:**
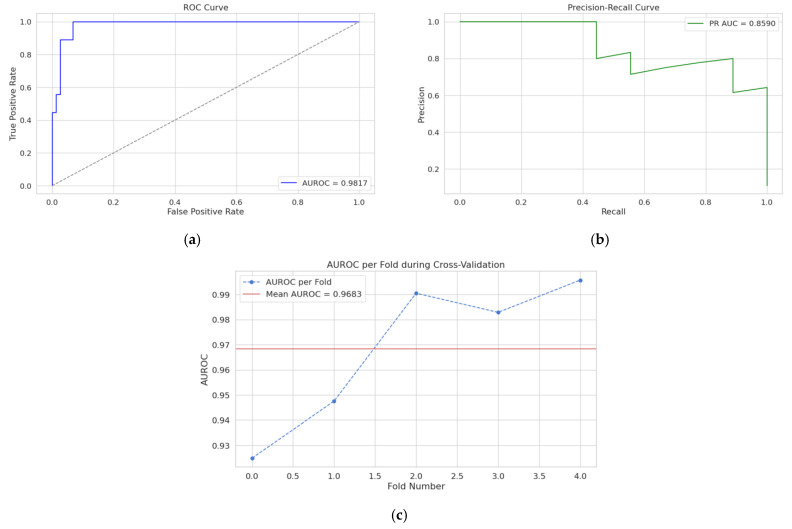
Performance of the BRF model for ARDS prediction within polytrauma patients. (**a**) ROC curve for the held-out test set. (**b**) PR-AUC for the held-out test set. (**c**) Cross validation AUROC distribution.

The BRF model demonstrated exceptional predictive performance across all evaluation metrics. Table 2 presents the comprehensive performance assessment on the holdout test set, showing AUROC of 0.98, precision of 0.80, recall (sensitivity) of 0.91, specificity of 0.80, F1-score of 0.84, and MCC of 0.70. The precision–recall AUC reached 0.86, confirming robust performance despite class imbalance. Figure 3 presents the receiver operating characteristic curves and precision–recall curves demonstrating excellent discrimination ability. During stratified 5-fold cross-validation, the model showed consistent performance, with a mean AUROC of 0.96 ± 0.02 (range: 0.93–0.99) across folds, indicating excellent stability and generalizability.

### 3.8. Model Interpretability and Clinical Insights

**Figure 4:**
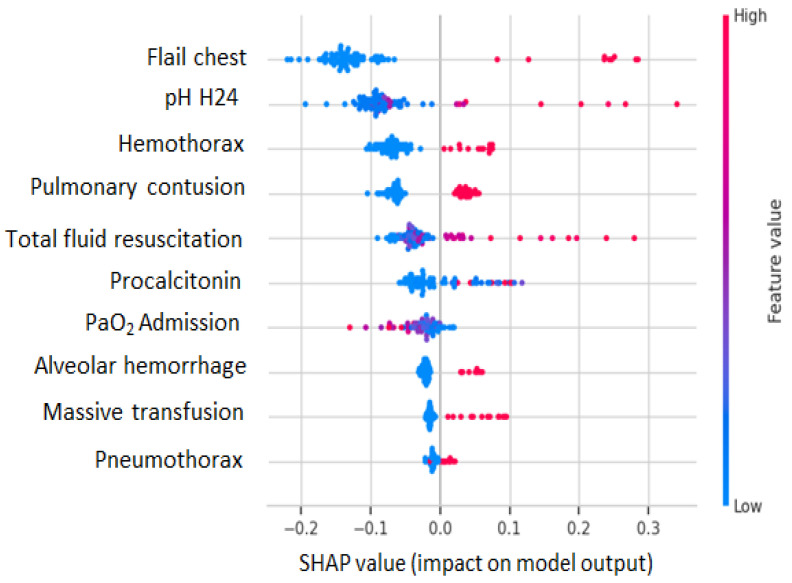
SHAP-based feature importance for ARDS prediction in polytrauma patients.

SHAP analysis provided detailed insights into the contributions of features to model predictions. Figure 4 presents the SHAP summary plot showing that flail chest injury, 24-h pH, and hemothorax had the most substantial impact on model predictions. Higher procalcitonin values, lower PaO_2_ levels, and the presence of thoracic injuries consistently increased ARDS prediction probability. Interestingly, higher 24-h pH values were associated with increased ARDS risk, contrasting with conventional expectations but consistent with early respiratory alkalosis observed in ARDS development. The model’s interpretability enables clinicians to understand the rationale behind the prediction, thereby enhancing clinical acceptability and supporting informed decision-making.

## 4. Discussion

### 4.1. Principal Findings and Clinical Significance

The BRF model demonstrated exceptional performance in predicting the development of ARDS in polytrauma patients, confirming our primary hypotheses regarding the effectiveness of comprehensive ML approaches for this challenging clinical prediction task. The AUROC of 0.98 represents a substantial improvement over traditional prediction methods and compares favorably with recent ML studies in ARDS prediction, which typically report AUROC values ranging from 0.65 to 0.87 [16,27,28]. The precision–recall AUC of 0.86 demonstrates robust performance despite severe class imbalance, addressing a critical limitation of many previous studies that failed to handle skewed datasets adequately. This suggests that the combination of Tomek Links undersampling, ANOVA feature selection, and SMOTE oversampling effectively preserves the underlying data distribution while enhancing the representation of the minority class.

### 4.2. Feature Importance and Clinical Interpretation

Procalcitonin emerged as the most discriminative feature (F-score: 4.89), consistent with its established role as a biomarker for sepsis and inflammatory conditions in critically ill patients. Patients with higher procalcitonin levels at admission were more likely to develop respiratory failure, consistent with previous studies showing a significant association between procalcitonin and ARDS [35,36,47]. The identification of procalcitonin as a key predictor underscores the inflammatory nature of ARDS pathogenesis and suggests potential therapeutic targets for early intervention. PaO_2_ at ICU admission (F-score: 4.73) was a crucial early indicator, with lower values predicting higher ARDS risk. This finding supports the clinical observation that initial oxygenation impairment often precedes full ARDS criteria fulfillment, providing an opportunity for early recognition and intervention.

### 4.3. Thoracic Injury Patterns and ARDS Risk

The prominence of thoracic injuries in our feature selection aligns with the established understanding of direct lung injury mechanisms in trauma-related ARDS. Flail chest injury demonstrated the strongest clinical association (89.13% in ARDS vs. 51.92% in non-ARDS, *p* < 0.001), consistent with several studies that have identified this condition as an independent risk factor for ARDS development [48,49]. The paradoxical chest wall movement associated with flail chest creates a ventilation–perfusion mismatch and can compromise pulmonary tissue integrity, predisposing patients to secondary inflammatory responses. Pulmonary contusion (86.95% in ARDS patients) and hemothorax (56.52% in ARDS patients) similarly represent direct mechanisms of lung injury that can trigger the inflammatory cascade leading to ARDS. These findings underscore the importance of thoracic trauma assessment in early ARDS risk stratification and suggest that patients with multiple thoracic injuries require heightened surveillance and potentially preemptive interventions.

### 4.4. Fluid Management and Transfusion-Related Risk Factors

Higher vascular filling emerged as a prominent distinguishing feature, with ARDS patients receiving significantly more crystalloids and colloids during the first 24 h after admission (3594 ± 1967 vs. 2055 ± 1604 mL, *p* = 0.025). Overzealous crystalloid administration can induce capillary leakage associated with pulmonary dysfunction through neutrophil activation, supporting conservative fluid management strategies in high-risk patients [50]. Massive transfusion (F-score: 4.12) represented another critical risk factor, occurring in 34.78% of ARDS patients compared to 7.69% of non-ARDS patients. This association is well-established in the literature, with massive transfusion potentially leading to Transfusion-related Acute Lung Injury through both immunologic and non-immunologic pathways [51,52]. The identification of these modifiable risk factors suggests potential targets for preventive interventions, including restrictive transfusion protocols and balanced fluid resuscitation strategies.

### 4.5. Acid-Base Physiology and Early ARDS Recognition

The counterintuitive finding that higher 24-h pH values are associated with increased ARDS risk (F-score: 4.35) provides essential insights into the early pathophysiology of ARDS. This finding contrasts with conventional expectations that acidosis is associated with worse outcomes but aligns with observations of early respiratory alkalosis caused by tachypnea and hyperventilation in the initial phases of ARDS [53]. Early respiratory alkalosis may signal aggressive compensatory mechanisms or reflect specific ventilator settings used in response to developing respiratory insufficiency. This finding highlights the complexity of acid-base physiology in critically ill patients and demonstrates the potential of ML to identify subtle patterns that might escape traditional clinical recognition.

### 4.6. Surgical Interventions and the “Second Hit” Phenomenon

In our cohort, emergency surgical operations were recorded with a median delay of 6 h (IQR 2–18 h; mean 17.4 ± 46.7 h) from hospital admission, illustrating the clinical evidence of polytrauma care, with the majority of surgical procedures performed early after primary resuscitation. In contrast, a minority were delayed due to unstable clinical conditions or transfer requirements. Although neurological, digestive, and orthopedic surgeries were more prevalent in the ARDS group, undergoing emergency interventions in general did not appear among the top predictors in our BRF model. These findings suggest that early surgical interventions may not act as a predominant “second hit” triggering ARDS in this cohort. Although this pattern aligns with contemporary trauma principles, including Early Appropriate Care (EAC) [54] and Damage Control Orthopaedics (DCO) [55], we cannot definitively determine whether the timing was protocol-driven or dependent on patient condition or inter-hospital transfer logistics. Nevertheless, the predominance of early surgical interventions may partly explain why emergency surgery did not appear as a consistent independent predictor of ARDS in our model. Instead, the main drivers of ARDS in this population are likely thoracic trauma patterns and early physiological insults captured by our ML model. In highly injured patients, the development of ARDS is often shaped not only by the primary traumatic insult (or first hit), but also by following physiological stressors, including surgery, a phenomenon frequently conceptualized as a “second hit” [56]. This latter triggers systemic inflammatory responses marked by the secretion of pro-inflammatory cytokines, including IL-6, IL-8, and TNF-α, which induce endothelial activation, neutrophil infiltration, and capillary permeability, thereby increasing the risk of complications such as systemic inflammatory response syndrome (SIRS), multiorgan failure, and ARDS [57]. This notion is thoroughly documented in trauma literature: timely definitive fixation of long-bone fractures in critically ill patients, or combined emergent operation, can lead to a marked inflammatory response, resulting in ARDS and multiorgan failure [58]. Neurosurgical interventions may also mediate through a brain-lung “double-hit ”mechanism, characterized by cytokine release following severe head injury, which prepares the lungs for a secondary insult [56]. The concepts of Early Appropriate Care (EAC) and Damage Control Orthopaedics (DCO) were established primarily to reduce this risk by prolonging high-burden surgeries once stabilization is achieved [54,55].

### 4.7. Comparison with Previous Studies and Model Performance

Our BRF model’s performance compares favorably with recent ML applications in ARDS prediction. A German study using radiomic features from chest CT scans achieved an AUROC of 0.79 with a recall and precision of 0.80 and 0.76, respectively [59]. Wang and colleagues evaluated multiple algorithms for ARDS prediction in traumatic brain injury patients, with random forest achieving an AUROC of 1.0 in training but only 0.652 in validation, suggesting overfitting issues [20]. Christie and colleagues developed a SuperLearner ensemble model for trauma outcomes, including ARDS, achieving an AUROC of 0.87, but with limited details on sensitivity, specificity, and handling of class imbalance [60]. Our systematic approach to addressing class imbalance, rigorous feature selection, and comprehensive validation methodology likely contributed to the superior and more generalizable performance observed in our study.

### 4.8. Clinical Implementation and Decision Support

The model’s interpretability, as facilitated by SHAP analysis, enhances clinical acceptance and implementation by providing transparent reasoning for predictions. Clinicians can understand which factors contribute most strongly to ARDS risk, enabling informed decision-making about monitoring intensity, preventive interventions, and resource allocation. The use of routinely available clinical parameters ensures practical implementation without requiring specialized testing or additional costs. The balanced approach to sensitivity (0.91) and specificity (0.80) provides clinically appropriate trade-offs, maximizing ARDS case detection while maintaining a reasonable false-positive rate. This balance is crucial in critical care settings, where missed diagnoses carry severe consequences but excessive false alarms can strain resources and lead to intervention fatigue.

### 4.9. Study Limitations and Future Directions

Several limitations must be acknowledged in interpreting our findings. The single-center retrospective design limits generalizability to other populations and healthcare systems with different patient characteristics, treatment protocols, and resource availability. Our work is constrained by the scarcity of detailed preoperative resuscitation protocols and intraoperative data, and the lack of systematic documentation of organizational-level changes in trauma management during the study period, which may affect the incidence of ARDS and temporal trends.

Regional variations in trauma patterns, patient demographics, and clinical practices may affect model performance when applied to different populations. The relatively small sample size of ARDS cases (n = 43), while adequate for ML model development, may limit the robustness of some findings and the ability to detect subtle associations. The retrospective design precludes assessment of temporal relationships and potential confounding factors that might influence both predictor variables and outcomes.

External validation across independent cohorts from different geographic regions and healthcare systems is the most critical next step to confirm model generalizability. Prospective validation studies would provide stronger evidence of clinical utility and enable assessment of real-world implementation challenges. Future research should explore the integration of additional data modalities, including continuous physiological monitoring data, advanced imaging features, and genomic biomarkers, to further enhance prediction accuracy. The development of real-time prediction systems that provide dynamic risk assessment as new clinical data become available is a vital area for future investigation.

### 4.10. Clinical and Policy Implications

The successful development of a high-performance ARDS prediction model has several important implications for clinical practice and healthcare policy. Early identification of high-risk patients enables proactive interventions that may prevent the development of ARDS or mitigate its severity, potentially improving outcomes and reducing healthcare costs. The model could support clinical decision-making regarding ICU admission, monitoring intensity, and resource allocation in resource-constrained environments. Implementing such prediction tools could facilitate the development of personalized treatment protocols tailored to individual risk profiles, thereby advancing precision medicine in critical care.

From a research perspective, the model could enhance clinical trial design by enabling more precise patient stratification and enrichment strategies. Identifying patients at the highest risk of developing ARDS could improve the efficiency of intervention studies and accelerate the development of effective preventive therapies. The methodology demonstrated in this study provides a framework for developing similar prediction tools for other critical care complications, potentially transforming the approach to risk assessment and prevention in intensive care medicine.

## 5. Conclusions

This study demonstrates the successful development and validation of a BRF ML model for early prediction of ARDS in critically ill polytrauma patients. The comprehensive methodology, incorporating Tomek Links undersampling, ANOVA-based feature selection, and SMOTE oversampling, effectively addressed class imbalance while maintaining exceptional predictive performance. The model achieved an AUROC of 0.98, a sensitivity of 0.91, and a specificity of 0.80, representing substantial improvement over traditional prediction approaches and comparing favorably with recent ML studies in critical care. The identification of ten key predictive features, including procalcitonin, PaO_2_, 24-h pH, massive transfusion, fluid resuscitation volume, and specific thoracic injuries, provides clinically interpretable insights that can guide early intervention strategies and resource allocation decisions.

The robust performance across multiple evaluation metrics, including a PR-AUC of 0.86 and an MCC of 0.70, confirms the model’s ability to handle imbalanced datasets effectively. The consistent performance across stratified cross-validation (AUROC range: 0.93–0.99) indicates excellent stability and strong generalization. These findings support integrating ML approaches into critical care decision-making, offering clinicians powerful tools for early recognition and intervention in ARDS. The use of readily available clinical parameters ensures practical implementation without requiring additional diagnostic testing or specialized equipment.

This research is constrained by a limited sample size of 43 ARDS cases, which increases the risk of overfitting. As highlighted by Riley et al. [30,31], small-event prognosis studies may produce a sensitive coefficient and inaccurate predictions. Although formal sample-size calculations were not applicable retrospectively, we implemented the methods described above to address these limitations.

External validation in diverse populations and healthcare settings remains essential for confirming generalizability and supporting the widespread clinical implementation of findings. Future research should focus on prospective validation studies, real-time implementation assessment, and exploration of additional data modalities to further enhance prediction accuracy. The methodology and findings presented in this study provide a foundation for developing similar prediction tools for other critical care complications, potentially transforming risk assessment and preventive care in intensive care medicine.

## Figures and Tables

**Table 1:** Baseline characteristics of polytraumatic patients with or without ARDS and missingness rate.

Variables		% Missingness	ARDS (n = 43)	Non-ARDS (n = 392)	*p*-Value
Demographics					
Age		0%	44 ± 16.52	40 ± 16.52	0.064
Gender	Male	0%	40 (93%)	343 (84.85%)	0.18
	Female		3 (7%)	49 (13.22%)	
Medical History					
Asthma		0%	2 (4.3%)	2 (0.48%)	<0.001
Tabac		0%	27 (58.7%)	195 (46.87%)	0.148
COPD		0%	7 (15.2%)	6 (1.44%)	<0.001
Alcoholism		0%	5 (10.9%)	92 (22.11%)	0.049
Drug consumption			1 (2.2%)	17 (4.08%)	0.516
ASA classification	ASA 1	0%	20 (47.82%)	274 (69.89%)	0.16
	ASA 2	0%	21 (50%)	114 (29%)	
	ASA 3	0%	1 (2.17%)	4 (1.02%)	
Injury Assessment					
Severe isolated brain injury		0%	2 (4.3%)	57 (13.7%)	1.48
Axial trauma		0%	24 (52.2%)	111 (26.68%)	<0.001
Abdominal trauma		0.24%	22 (47.8%)	128 (30.76%)	0.023
Pelvic trauma		0.24%	8 (17.4%)	34 (8.17%)	0.044
Pneumothorax		0.24%	21 (45.65%)	63 (15.14%)	<0.001
Alveolar Hemorrhage		0.24%	15 (32.6%)	40 (9.61%)	<0.001
Flail chest injury		0.24%	41 (89.13%)	216 (51.92%)	<0.001
Pulmonary contusion		0.24%	40 (86.95%)	169 (40.62%)	<0.001
Hemothorax		0.24%	26 (56.52%)	75 (18.02%)	<0.001
Long bone fracture		0.24%	10 (21.73%)	62 (14.9%)	0.240
Trauma Severity Scores					
ISS		0%	35 ± 13.92	25.87 ± 15.16	<0.001
TRISS		0%	29.28 ± 26.26	20.95 ± 18.16	0.17
RTS		0%	6.55 ± 1.41	6.43 ± 2.51	0.67
Emergency Procedures					
Emergency intubation		0%	28 (60.86%)	265 (63.7%)	0.629
Tranexamic acid		0%	29 (63.04%)	159 (38.22%)	0.001
Catecholamines		0.24%	38 (82.60%)	250 (60.09%)	0.002
Fibrinogen		4.16%	12 (26.08%)	40 (9.61%)	<0.001
Total vascular filling/24 h (mL)		0.5%	3594 ± 1967	2055 ± 1604	0.025
Massive transfusion		0.24%	16 (34.78%)	32 (7.69%)	<0.001
Transfusion		0%	32 (69.56%)	152 (36.53%)	<0.001
Emergency surgery					
Neurosurgery		2.6%	5 (11.63%)	37 (10.16%)	0.025
Digestive surgery		0%	5 (11.63%)	25 (6.87%)	0.038
Thoracotomy		0%	1 (2.33%)	5 (1.37%)	0.574
Vascular surgery		0%	1 (2.33%)	5 (1.37%)	0.589
Orthopedic surgery		0%	12 (27.91%)	49 (13.46%)	0.007
Clinical data upon admission					
Respiratory rate (cycles/min)		0%	30 (21.5; 35)	22 (18; 29)	0.14
SPO_2_ (%)		21.62%	90.28 ± 6.49	92.93 ± 6.27	<0.001
GCS		0%	11.48 ± 4.19	9.89 ± 4.41	0.212
Laboratory data upon admission					
Hemoglobin (g/dL)		0%	10.55 ± 2.57	11.47 ± 2.49	0.19
Hematocrit (%)		0%	31.4 ± 6.88	33.30 ± 6.85	0.41
PCT (g/L)		13.26%	2.8 (0.26; 7.51)	0.18 (0.05; 1.03)	0.035
CRP		12%	118 ± 112	95.91 ± 79.86	0.001
Creatinine (mmol/L)		0%	110 ± 60.31	80.2 ± 64.12	0.002
White blood cells (10^3^/mm^3^)		0%	19.37 ± 8.22	16.28 ± 6.70	0.008
Prothrombin time (%)		0.24%	59.76 ± 19.74	72.01 ± 19.28	<0.001
Platelets (10^3^/mm^3^)		0%	203.93 ± 74.75	200.83 ± 71.07	0.66
Arterial blood gases upon admission					
pH		7.12%	7.30 ± 0.12	7.36 ± 0.11	0.002
PaO_2_ (mmHg)		7.12%	136.34 ± 8.46	190.6 ± 104.05	0.03
PaCO_2_ (mmHg)		7.12%	40.87 ± 13.83	35.75 ± 9.17	0.001
FiO_2_/PaO_2_ ratio		13.76%	98.93 ± 64.81	328.87 ± 133.4	0.001
HCO_3_^−^ (mmol/L)		7.12%	19.14 ± 5.4	19.95 ± 4.01	0.334
Lactate (mmol/L)		2.7%	4.35 ± 3.41	3.02 ± 2.36	0.15

ARDS: Acute respiratory distress syndrome; COPD: Chronic obstructive pulmonary disease; ASA: American Society of Anesthesiologists classification; ISS: Injury severity score; TRISS: Trauma and Injury Severity Score; RTS: Revised trauma score; SpO_2_: Oxygen saturation; GCS: Glasgow Coma Scale; PaO_2_: Partial pressure of oxygen; PaCO_2_: Partial pressure of carbon dioxide; FiO_2_: Inspired fraction of oxygen; HCO_3_**^−^**: Bicarbonate ions; CRP: C-reactive protein; PCT: Procalcitonin.

**Table 2:** Performance metrics of the balanced random forest classifier on the test set.

Metric	Score
AUROC	0.98
PR-AUC	0.86
MCC	0.70
Precision	0.80
Recall	0.91
F1 score	0.84

AUROC: Area Under the Receiver Operating Characteristic; PR-AUC: Precision–Recall Area Under the Curve; MCC: Matthews Correlation Coefficient.

## Data Availability

The data supporting the findings of this study are available from CTGB Hospital; however, restrictions apply due to patient confidentiality and institutional regulations, and therefore, the data are not publicly available. However, the authors have data available upon reasonable request, provided prior approval is obtained from the CTGB Hospital Ethical Committee.
